# Morphological and Functional Characterization of IL-12Rβ2 Chain on Intestinal Epithelial Cells: Implications for Local and Systemic Immunoregulation

**DOI:** 10.3389/fimmu.2018.01177

**Published:** 2018-05-29

**Authors:** Mari Regoli, Angela Man, Nadhezda Gicheva, Antonio Dumont, Kamal Ivory, Alessandra Pacini, Gabriele Morucci, Jacopo J. V. Branca, Monica Lucattelli, Ugo Santosuosso, Arjan Narbad, Massimo Gulisano, Eugenio Bertelli, Claudio Nicoletti

**Affiliations:** ^1^Department of Molecular and Developmental Medicine, University of Siena, Siena, Italy; ^2^Gut Health Programme, Quadram Institute Bioscience, Norwich, United Kingdom; ^3^European Food Safety Authority, EFSA, Parma, Italy; ^4^Department of Experimental and Clinical Medicine, University of Florence, Florence, Italy

**Keywords:** IL12Rβ2 chain, intestinal epithelial cell, immunoregulation, mucosal immunity, interleukin 12

## Abstract

Interaction between intestinal epithelial cells (IECs) and the underlying immune systems is critical for maintaining intestinal immune homeostasis and mounting appropriate immune responses. We have previously showed that the T helper type 1 (T_H_1) cytokine IL-12 plays a key role in the delicate immunological balance in the gut and the lack of appropriate levels of IL-12 had important consequences for health and disease, particularly with regard to food allergy. Here, we sought to understand the role of IL-12 in the regulation of lymphoepithelial cross talk and how this interaction affects immune responses locally and systemically. Using a combination of microscopy and flow cytometry techniques we observed that freshly isolated IECs expressed an incomplete, yet functional IL-12 receptor (IL-12R) formed solely by the IL-12Rβ2 chain that albeit the lack of the complementary IL-12β1 chain responded to *ex vivo* challenge with IL-12. Furthermore, the expression of IL-12Rβ2 on IECs is strategically located at the interface between epithelial and immune cells of the lamina propria and using *in vitro* coculture models and primary intestinal organoids we showed that immune-derived signals were required for the expression of IL-12Rβ2 on IECs. The biological relevance of the IEC-associated IL-12Rβ2 was assessed *in vivo* in a mouse model of food allergy characterized by allergy-associated diminished intestinal levels of IL-12 and in chimeric mice that lack the IL-12Rβ2 chain on IECs. These experimental models enabled us to show that the antiallergic properties of orally delivered recombinant *Lactococcus lactis* secreting bioactive IL-12 (rLc-IL12) were reduced in mice lacking the IL-12β2 chain on IECs. Finally, we observed that the oral delivery of IL-12 was accompanied by the downregulation of the production of the IEC-derived proallergic cytokine thymic stromal lymphopoietin (TSLP). However, further analysis of intestinal levels of TSLP in IL-12Rβ2^−/−^ mice suggested that this event was not directly linked to the IEC-associated IL-12Rβ2 chain. We interpreted these data as showing that IEC-associated IL12Rβ2 is a component of the cytokine network operating at the interface between the intestinal epithelium and immune system that plays a role in immune regulation.

## Introduction

The constant dialog between the intestinal epithelium and underlying immune system plays a key role in intestinal immune homeostasis by shaping the appropriate immunological microenvironment (1). Intestinal epithelial cells (IECs) signal to the immune system *via* the secretion of regulatory cytokines; in the presence of commensal bacteria, IECs secrete anti-inflammatory molecules that include thymic stromal lymphopoietin (TSLP) (2) and transforming growth factor-β (3). By contrast, pathogens cause epithelial cells to release proinflammatory factors, such as IL-8 (CXCL-8), MCP-1 (CCL2) and MIP-3α (4, 5) and might trigger rapid response to clear the infection (6). In turn, cytokines produced by the intestinal immune system may signal back to the epithelium and regulate epithelial functions such as, for example, antigen sampling by specialized M cell (7). The T helper type 1 (T_H_1) cytokine IL-12 plays a variety of roles in intestinal immunity ranging from response to pathogens (8), interaction with group 1 innate lymphoid cells in maintaining barrier integrity (9) to protecting against food allergy (10). With regard to the latter we have previously shown that the intestinal levels of IL-12p70 in the gut are critical for the resistance to food allergy (10). Indeed, sensitization to the food allergen peanuts was characterized by significantly reduced intestinal levels of IL-12. One of the most convincing evidence for the regulatory role of IL-12 in the intestinal mucosa was brought about by the observation that blocking IL-12 in the gut at the time of antigen presentation by targeted oral delivery of microencapsulated neutralizing antibody significantly increased susceptibility to food allergy in mice (10). The notion that IL-12 is critical in food allergy is further strengthened by the observation that oral delivery of liposome encapsulated IL-12 suppressed allergic reaction (11). However, aspects of its mechanism(s) of action remained to be determined. We observed that IECs expressed only the low affinity IL-12 binding site IL-12Rβ2 chain, but not the complementary high affinity binding site IL-12Rβ1 chain. IEC-associated IL-12Rβ2 operates as an incomplete, yet still operational IL12R the expression of which is under the control of immune cell-derived signals. This observation prompted us to test the hypothesis that the presence of IEC-associated IL-12Rβ2 could directly affect the regulatory properties of IECs. This was done by adopting a strategy based on oral administration of a *Lactococcus lactis* strain genetically engineered to produce biologically active IL-12 (rLc-IL12) (12) in allergic wild-type (wt) and chimeric mice that lacked IL-12Rβ2 on the intestinal epithelium. We observed that the lack of IL-12Rβ2 on IECs significantly reduced the antiallergy activity of rLc-IL12. Also, IL-12 mediated suppression of food allergy was accompanied by the downregulation of the IEC-derived cytokine TSLP.

These data suggested that IEC-associated IL-12Rβ2 contributed to control the finely tuned regulatory cytokine network operating at the lymphoepithelial interface in the gut and impacted on the host’s ability to mount appropriate immune responses.

## Materials and Methods

### Mice and Bacterial Strain

Mice were maintained in an access-restricted room in conventional conditions. B6.129S1-Il12rb2tm1Jm/J (IL-12Rβ2^−/−^) and wt C57BL/6J mice that were previously used to generate chimeric mice (13) were purchased from (Jackson Laboratory, Bar Harbor, ME, USA). IL-12Rβ2^−/−^ mice were maintained on BL/6 background. Chimeric mice were produced by adopting a slightly modified busulfan-based method that allowed a stable bone marrow (BM) chimerism (14). Briefly, recipient mice were treated with daily injections of 20 mg/kg busulfan (6 consecutive days for a total dose of 120 mg/kg busulfan). Twenty-four hours after the last BU injection, 5 × 10^6^ BM cells collected from the femur and tibia of wt or IL-12Rβ2^−/−^ donors and injected i.v. (tail vein) into recipient mice. Age- and sex-matched busulfan-treated IL12Rβ2-deficient mice were reconstituted either with BM from wt mice (WT→ IL12Rβ2^−/−^) to generate mice lacking IL-12Rβ2 in IECs (IL-12Rβ2^∆IEC^) or BM from syngeneic IL-12Rβ2-deficient mice (IL-12Rβ2^−/−^ → IL-12Rβ2^−/−^) to control for the effects of the busulfan treatment and BM reconstitution. Mice were then allowed to rest for at least 8 weeks before the beginning of the experiments. Chimerism was functionally assessed by determining the ability to produce IFNγ *in vivo* following i.p. administration of mouse IL-12 (1 μg/mouse for 5 consecutive days) as previously described (15). Furthermore, wt C57BL/6J and chimeric mice were sensitized according to a well-established model of food (peanuts) allergy (10, 16). Ground whole peanuts were used as antigen and crude peanut extract (CPE) was prepared as described in detail previously (10, 17). Briefly, mice received intragastric (i.g.) administration (oral gavage) of 1 mg/mouse of ground whole peanut together with 10 µg of cholera toxin (CT) (Quadratech Ltd., Surrey, UK) in PBS on days 0, 7, 14, 21 and 28. On day 35, all mice were challenged by oral gavage with CPE (1 mg/mouse divided into two doses at 30 min intervals). The administration regimen of the sensitizing mixture was based on previously established protocol (10, 16). To assess the role of rLc-IL12 in suppressing food allergy, wt and chimeric mice were gavaged with PBS (untreated) or *L. lactis* FI10611 secreting bioactive IL-12p70 (rLc-IL12) (8–10 mice/group). Description of the wt *L. lactis* strain, generation of the recombinant IL-12 producing strain, levels and regulation of IL-12 secretion and its biological activity were described by us in detail previously (12). Strains were grown to an OD_600_ of approx 1.0. Cell pellets were collected, washed with PBS and resuspended in 2% NaHCO_3_. A suspension of 100 µl containing 10^9^ colony-forming units/ml was administrated by oral gavage in a single dose to the mice 24 and 2 h before administration of the sensitizing mixture. The percentage of mice that developed Type I hypersensitivity reaction was assessed as previously described (10). Serum levels of peanut-specific IgE antibody were determined by ELISA in all groups of sensitized and control mice as described previously in detail (18). Plasma levels of histamine were detected by using an enzyme immunoassay kit (Immunotech S.A.S., Marseille, France) whereas the vascular leakage was monitored by measuring the hematocrit. Body temperature was monitored using a rectal probe (Kent Scient. Corp. Torrington, USA). Levels of the cytokines IL-4 and IL-5 produced by splenocytes *in vitro* following antigen (CPE 20 µg/ml) recall challenge (72 h); plasma levels of IFNγ were determined by ELISA kits (ELISA MAX™ Deluxe Sets, Biolegend-Cambridge Bioscience, Cambridge, UK).

### Gene Expression

Total RNA was extracted from gut tissues using RNeasy Mini Kit (Qiagen Ltd.). For cDNA synthesis, total RNA was reverse transcribed with Quantitect Reverse Transcription Kit (Qiagen Ltd). Q-PCR was performed on 5 µl of diluted (1:5) template cDNA, using the ABI 7500 Real-Time PCR System (Applied Biosystems, Warrington, UK) and SYBR Green Detection. Data were normalized against an invariant endogenous control, 18S ribosomal RNA, and threshold cycle number (Ct) values obtained were converted into fold of relative induction using the ΔΔCt. The following primers were used: 18s: F CGG ACA GGA TTG ACA GAT TG-3′, R 5′-CAA ATC GCT CCA CCA ACT AA; OX40L: F: CCC TCC AAT CCA AAG ACT; R: ATC CTT CGA CCA TCG TTC; TSLP: F:CCG ATG GGG CTA ACT TAC; R: TCC TGA TTT GCT CGA ACT; IL-33: F: GAT GGG AAG AAG CTG ATG GTG; R: TTG TGA AGG ACG AAG AAG.

### Immunohistochemistry and Transmission Electron Microscopy (TEM)

Frozen sections (8–10 µm) were fixed with 10% buffered formalin for 30 min and endogenous peroxidases were quenched with 0.3% hydrogen peroxide for 1 h and stained with biotinylated goat anti-mouse TSLP (R&D Systems) and then treated with Vectastain Elite ABC kit (Vector Laboratories). Fluorescein deposit was achieved on the site of the immunoreaction with the TSA Plus fluorescence system (Perkin-Elmer). Sections were counterstained with mouse anti-β-actin Mab (Sigma-Aldrich, Milan) followed by TRITC-conjugated donkey anti-mouse IgG antibody (Jackson ImmunoResearch Lab-Starfish, Milan, Italy). For TEM analysis, tissues were fixed in 4% paraformaldehyde and, following dehydration, embedded in Epon 812. Sections were incubated overnight at 4°C with rabbit anti-IL12Rβ2 (Bioss Inc., Boston, MA, USA) followed by 15 nm gold-conjugated anti-rabbit IgG (BB International, Cardiff, UK) and examined with a 201 Philips TE microscope.

### Isolation of IECs From Mouse Small Intestine and Evaluation of IL-12R Expression and Functional Integrity

Mice were sacrificed; small intestines were removed, dissected longitudinally and washed with sterile PBS. Tissue was cut into 0.5 cm pieces and washed with sterile PBS. To remove IECs, the tissue was subjected to a first wash in PBS containing 10% FCS, 1 mM DTT (20 ml), and a second wash in PBS containing 10% FCS, 15 mM EDTA (20 ml) at 37°C, 250 rpm for 15 and 20 min, respectively. Preceding each wash, tissue-buffer mix was vortexed for 1 min. After washings the resultant pellets were resuspended in PBS containing 10% FCS and stored on ice. CD45^+^ cells were removed from crude IEC preparations using magnetic labeling with Anti-PE Microbeads (Miltenyi Biotec) and magnetic separation with LD MidMACS depletion columns (Miltenyi Biotec). The expression and function of IL-12Rβ1 and IL-12β2 chains on viable IECs was assessed by flow cytometry. Briefly, isolated IECs were double-stained with anti-IL-12Rβ1-FITC and anti-IL-12Rβ2-PE or mouse IgG1-PE (R&D Systems) and mouse IgG1-FITC (eBiosciences, UK) isotype controls. After incubation for 1 h on ice, cells were washed once and pellets were resuspended and fixed with an equal volume of 4% formalin for 20 min at room temperature. Fixed cells were washed once with PBSA. The functional integrity of IL-12Rβ2 through MAPKp38 phosphorylation was tested as follows. Freshly isolated IECs were placed in a 37°C water bath in the presence of 500 µM H_2_O_2_ or 2 µg/ml IL-12 (Biolegend, UK) for various times. Control cells were left untreated. At the end of the incubation period, an equal volume of 8% buffered formalin was added. After 10 min, fixed cells were washed with PBSA and allowed to cool on ice; cells were then permeabilized on ice for at least 30 min with Perm Buffer III (BD Biosciences, UK). Cells were washed and stained for 30 min at room temperature with rabbit antiphospho-MAPKp38 (Cell Signaling Technology, Danvers, MA, US) or normal rabbit IgG (Senta Cruz Biotechnology, Dallas, TX, US) as control. Cells were then incubated for a further 30 min with donkey anti-rabbit IgG-FITC (Santa Cruz). For viability assessment cells were washed and resuspended in 1 ml PBS. Dead cells were stained using Live/Dead Fixable Violet Dead Cell Stain Kit (Life Technologies, UK) according to the manufacturer’s instructions and data were acquired by flow cytometry.

### Intestinal Organoids and Cell Culture

Small intestine organoids were produced according to Sato et al. (19). Crypts were counted and diluted to obtain a final concentration of 75,000 crypts/ml. Pelleted crypts were resuspended in Matrigel matrix (Corning) (1 ml/75,000 crypts) and 20 µl were seeded per well of a preheated (37°C) 48-well tissue culture plate (Greiner Bio One). Organoids were grown at 37°C and 5% CO_2_ and media was replaced daily. The media used were as follows. Small intestinal culture condition (ENR) (19) medium: 18.4 ml advanced DMEM/F12^+++^, 0.02% (vol/vol) B27 supplement (Invitrogen), 0.01% (vol/vol) N2 supplement (Invitrogen), n-acetylcysteine (final conc. 1.25 mM, Sigma-Aldrich), recombinant mouse epidermal growth factor (final conc. 50 ng/ml, Invitrogen), recombinant mouse Noggin (final conc. 4 ng/ml, Peprotech), recombinant mouse R-spondin (final conc. 1 ng/ml, R&D systems) and ROCK kinase inhibitor, y-27632 (final conc. 10 μM, Sigma- Aldrich). Advanced DMEM/F12^+++^ media: 500 ml advanced DMEM/F12 (Invitrogen), 1% (vol/vol) Glutamax 100× (Invitrogen), HEPES (final conc. 5 mM, Invitrogen) and penicillin (final conc. 100 U/ml)/streptomycin (final conc. 100 µg/ml, Invitrogen). In some experiments, conditioned medium (CM) from 3-day culture of Peyer’s Patch (PP)-derived immune cells was used as part of the culture medium for the development of the organoids. Passaging was done after 7 days by breaking the Matrigel matrix dome with a 1 ml pipette. The organoids were dissociated from the matrix dome by pipetting with a 200 µl pipette. Organoids were centrifuged (600 rcf for 5 min), resuspended into fresh Matrigel (ratio 1:3) and seeded as described above. MODE-K cells (20) were used to study the influence of immune derived-signals on the expression of IL-12Rβ2 on IECs. MODE-K cells were seeded in the top compartment of Transwells culture system (Costar) at 1 × 10^5^ cells/well. After 4 days of growth PP-derived lymphocytes were added to the culture. Lymphocytes were isolated from PPs by mashing between frost edged sterile glass slides and filtered through strainer. Following the scraping of the MODE-K monolayer, PP-lymphocytes and MODE-K cells were seeded together to the top compartments of the Transwells to enable cell-to-cell contact. For non-contact coculture, 1 × 10^6^ PP-lymphocytes were added to the bottom compartments of the Transwell. Expression of IL-12Rβ2 was examined by flow cytometry after 72 h.

### Statistical Analysis

The animal study data were analyzed by (ANOVA) Wilcoxon rank test. The results for the gene expression data show the average relative fold of induction of IL-12Rβ2, TSLP, OX40L and IL-33. Data were analyzed using a Student’s *t*-test (two-tailed, equal variance) followed by application of the Bonferroni correction the level of which was set at *p* ≤ 0.0125. In either case, *p*-value of ≤0.05 was considered statistically significant. The results are expressed as mean ± SD.

## Results

### IECs Express the IL-12Rβ2 Chain but Not the Complementary IL-12Rβ1 Chain

**Figure 1:**
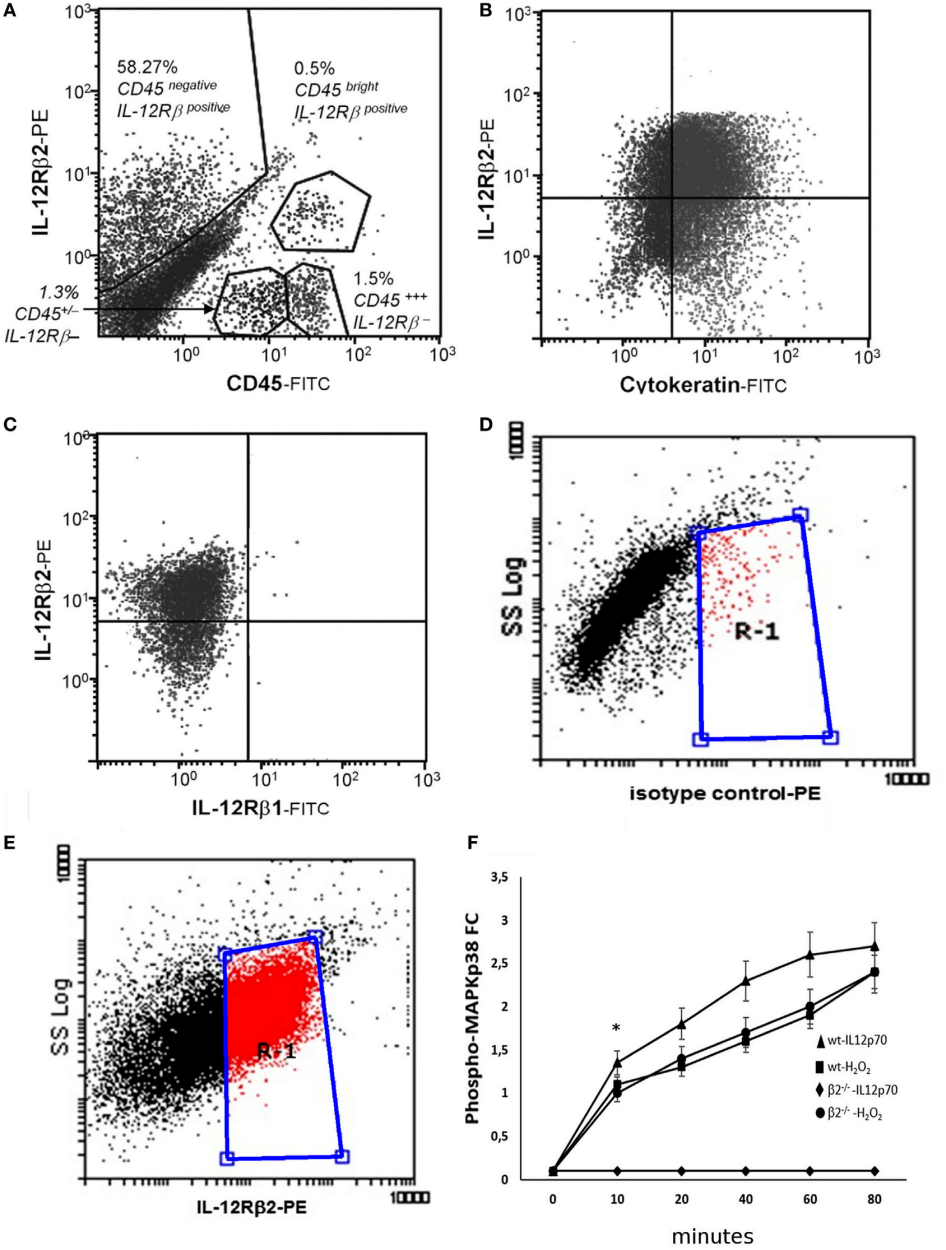
Expression and functional integrity of IL-12Rβ2 on intestinal epithelial cells (IECs). Flow cytometry analysis was carried out using epithelial cell suspension to identify intestinal cells expressing IL-12R. IL-12Rβ2 chain was detected on approximately 50% of small intestine CD45^−^ (IECs) cells and in a small proportion (>1%) of CD45^+^ cells **(A)**. IL-12Rβ2 positive cells that did not express CD45 (IL-12Rβ2^+^CD45^−^) were identified and selected. Their epithelial origin was confirmed through expression of cytokeratin **(B)**. Furthermore, we showed that the expression of IL-12Rβ2 on these cells was not paralleled by the presence of the complementary IL-12Rβ1 chain **(C)**. Also, plots of SSC vs. IL-12Rβ2 are shown **(D,E)**; Cells were stained with PE-coupled isotype control antibody to facilitate gating and enable detection of non-specific fluorescence **(D)**. Following staining with PE-coupled anti-IL-12Rβ2 antibody, positive cells were identified **(E)** within the gate R-1 constructed in D above. Finally, *ex vivo* stimulation of freshly isolated IECs from the mouse small intestine with IL-12p70 led to a rapid increase in intracellular phosphorylation of MAPKp38 only in IEC from wt mice but not IL-12Rβ2^−/−^ mice showing that the upregulation of phospho-MAPK38 is an IL-12Rβ2-specific event; stimulation with H_2_O_2_ was used as a positive control **(F)**. Experiments on MAPKp38 were performed in triplicate using freshly isolated IECs (five mice/group). (*) indicates statistical difference. *p* < 0.05.

The T_H_1 cytokine IL-12 binds to a heterodimeric IL-12Rβ1/β2 complexes on a variety of immune cells (21). First, flow cytometry analysis was carried out using epithelial cell suspension to identify intestinal cells expressing IL-12R. We observed that approximately 58% of small intestine CD45^−^ cells and in a small proportion (>1%) of CD45^+^ cells (Figure 1A) expressed IL-12Rβ2 chain. IL-12Rβ2 positive cells that did not express CD45 (IL-12Rβ2^+^CD45^−^) were identified and selected. The epithelial origin of the IL-12Rβ2^+^CD45^−^ cells was confirmed through expression of cytokeratin as seen in Figure 1B. Figure 1C demonstrated that these IL-12Rβ2^+^CD45^−^cytokeratin^+^ cells did not coexpress IL-12Rβ1. Side scatter (SSC) analysis is also shown (Figures 1D,E). This showed that the IL-12β2 chain is expressed *in vivo* by IECs in absence of the complementary IL-12Rβ1 chain.

**Figure 2:**
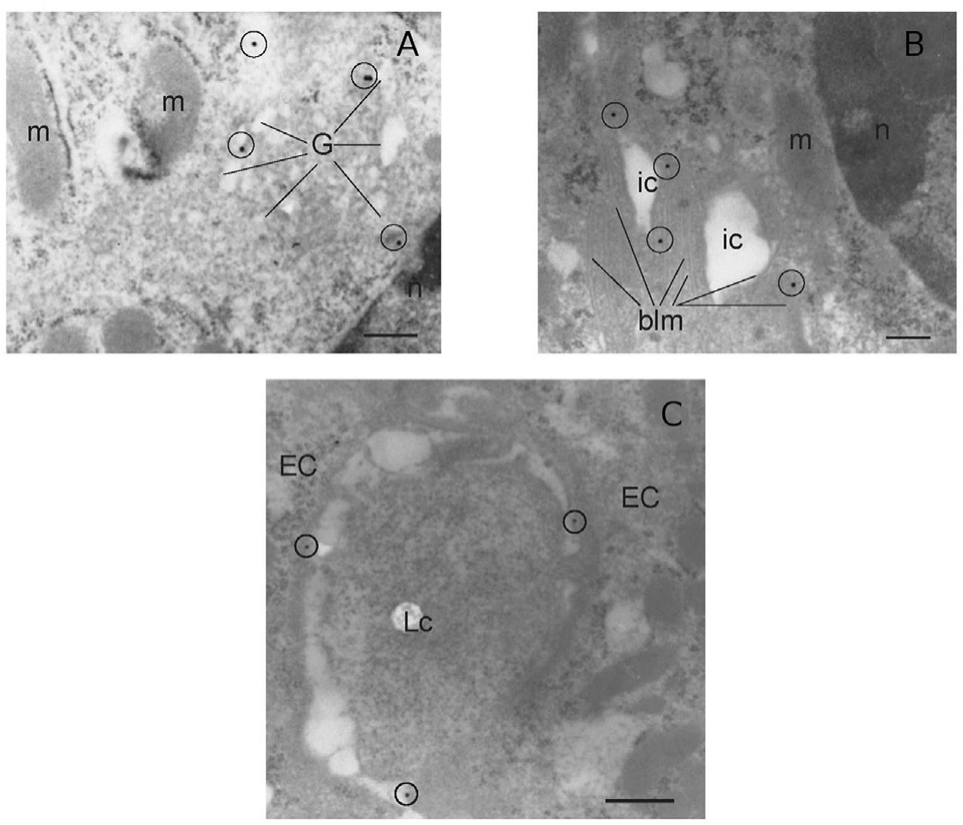
Morphological analysis of IL-12Rβ2 on IECs. Transmission electron micrograph analysis (immunogold staining) of mouse small intestine showed that IL-12Rβ2 in IECs was found in the Golgi **(A)** as well as the basolateral membrane **(B)** and at the interface between IECs and lymphoid cells **(C)**. G, Golgi; EC, epithelial cell; LC, lymphoid cell; m, mitochondria; n, nucleus; ic, intracellular space; blm, basolateral membrane; IECs, intestinal epithelial cells.

It has been shown that the presence of the IL-12Rβ2 chain is indispensable for IL-12 mediated signaling (15) although IL-12Rβ2 alone was insufficient to induce the canonical signal transducer and activator of transcription (Stat) 4 activation in response to IL-12 (22). However, an IL-12Rβ1/STAT4-independent pathway and based on MAP kinase (MAPK) activation in the presence of only the IL-12Rβ2 chain was observed (22). Thus, to assess the function of IL-12Rβ2 in the absence of the complementary IL-12Rβ1 chain, freshly isolated IECs were cultured in the presence of IL-12p70 and levels of phospho-MAPK38 were assessed. Rapidly, after the application of the stimulus, expression of phospho-MAPKp38 reached similar levels observed following challenge of IECs with hydrogen peroxide (H_2_O_2_) a non-specific activation stimulus (Figure 1F). By contrast, IECs isolated from IL-12Rβ2^−/−^ mice did not respond to challenge with IL-12p70 demonstrating that activation of MAPK38 was an IL-12Rβ2 specific event. The expression of the IL-12Rβ2 chain on IECs was further confirmed by TEM by immunogold staining. Positive staining was observed within the Golgi area (Figure 2A), in the proximity of the basolateral membrane (Figure 2B) and, most relevant to lympho-epithelial cross talk, at the interface between IECs and immune cells harboring the lamina propria (lp) (Figure 2C).

### The Expression of IL-12Rβ2 Chain on IECs Is Controlled by Immune System-Derived Signals

**Figure 3:**
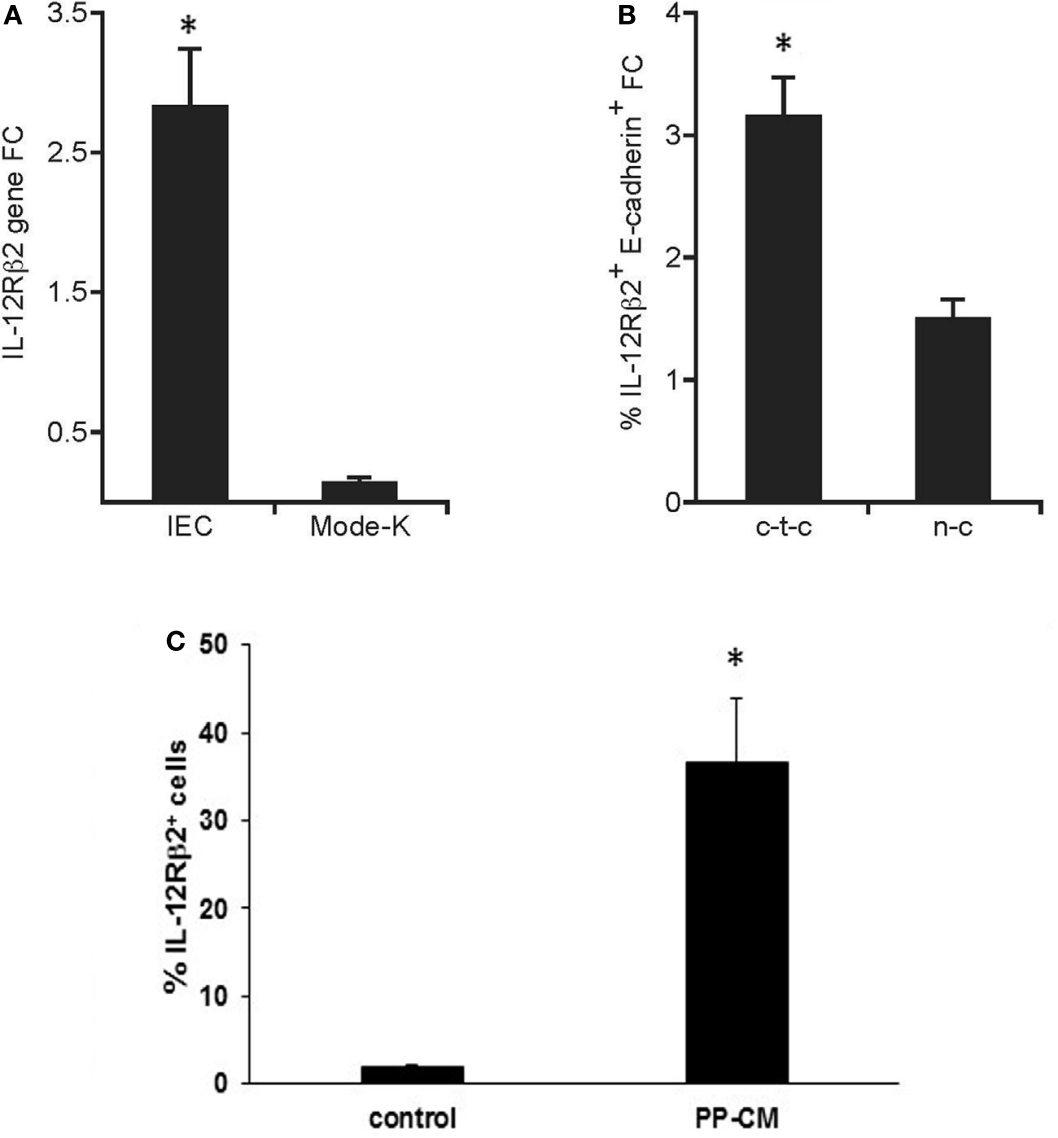
Immune system-derived signal(s) are required for the expression of functional IL-12Rβ2 on intestinal epithelial cells (IECs). Quantitative RT-PCR analysis showed that the expression of IL-12Rβ2 was significantly higher on freshly isolated primary IECs compared to cultured intestinal epithelial cell line MODE-K **(A)**. By contrast **(B)**, higher expression of IL-12Rβ2 (1.5-fold chance, FC, increase compared IL12Rβ2 expression levels in MODE-K untreated control cells) was seen when Peyer’s patch-derived lymphocytes were seeded in the bottom compartment of the Transwells culture system not in contact with MODE-K cells (n-c). However, a more pronounced increase (3.2-fold) was seen when PP-lymphocytes were allowed cell-to-cell interaction with the MODE-K cells (c-t-c). Expression of IL-12Rβ2 was also assessed on primary intestinal organoids developed in culture in the absence (control) or in presence of conditioned medium (CM) from 3-day cultures of PP-derived immune cells (PP-CM) chain **(C)**. A significant increase in the number of organoid cells expressed the IL-12Rβ2 was seen when the organoids were grown in the presence of CM. Transcript analysis was carried out in replicate mice (five mice/group) in two independent assays. Data were analyzed using a Student’s *t*-test (two-tailed, equal variance) followed by application of the Bonferroni correction the level of which was set at *p* ≤ 0.0125. Mode-K cells were pooled in two independent experiments and samples run in triplicate. Two batches of intestinal organoids/treatment were prepared and samples were run in triplicate. The results are expressed as mean ± SD. (*) indicates statistical difference. *p* < 0.05.

The *in vivo* observation that IL-12Rβ2 was expressed by IECs at the interface with local lp-immune cells prompted us to assess whether its expression was under the control of immune-derived signals. Quantitative RT-PCR analysis showed that the IL-12Rβ2 was expressed on freshly isolated primary IECs; however, its expression was negligible on the murine IEC line MODE-K (Figure 3A). By contrast, when using coculture systems (Figure 3B) we observed that PP-lymphocytes seeded in the bottom compartment of Transwells culture system induced the expression of the IL-12Rβ2 in MODE-K (1.5-fold increase). Yet, a more pronounced increase (3.2-fold) was seen when PP-lymphocytes were allowed cell-to-cell interaction with the MODE-K cells. The importance of immune-derived signals was further investigated in primary small intestine organoids that were developed *in vitro*. In the complete absence of immune cell-derived signals organoids IECs lacked the expression of the IL-12Rβ2 chain, however, when the organoids were grown in the presence of PP-derived conditioned medium (PP-CM) significantly higher number of IL-12Rβ2^+^ cells were observed (Figure 3C; Figure S1 in Supplementary Material). This experimental approach also showed that in the presence of PP-CM intestinal organoids also tend to increase the expression of cytokeratin (Figure S1 in Supplementary Material). Taken together these data demonstrated that cross-talk between IECs and immune cells is instrumental to trigger the expression of the IL-12Rβ2 on IECs.

### IEC-Associated IL-12Rβ2 Chain Plays a Role in Lympho-Epithelial Cross Talk

**Figure 4:**
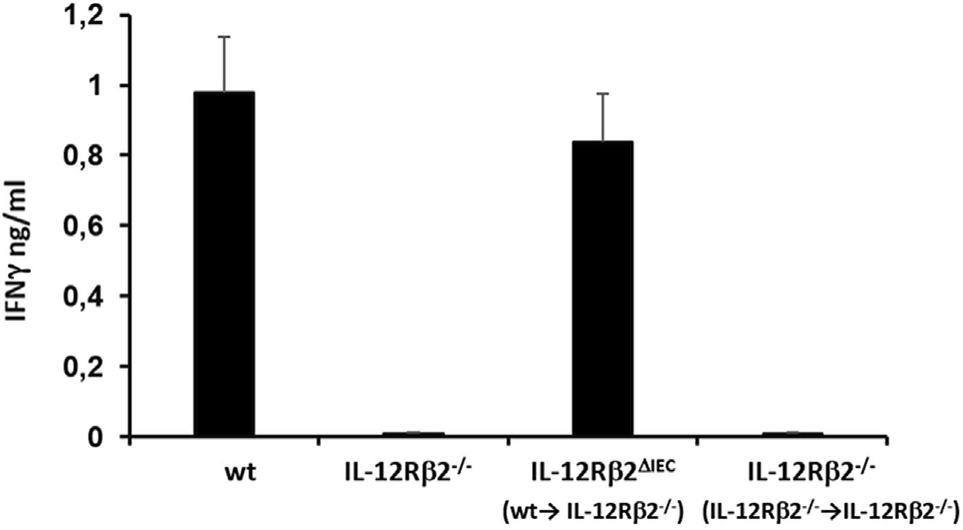
Bone marrow (BM) transplant restored *in vivo* IL-12-mediated production of IFNγ in IL-12Rβ2^−/−^ mice. Systemic (i.p.) administration of IL-12 induced the production of IFNγ by immune cells *via* IL-12/12R ligation. This response was absent in IL-12Rβ2^−/−^ mice; the pattern was different in chimeric mice generated by BM transplant from wild-type (wt) mice into IL-12Rβ2^−/−^ mice. At week 8 posttransplant the recipient mice that lacked IL-12Rβ2 exclusively in intestinal epithelial cells (IECs) (IL-12Rβ2^∆IEC^) showed a physiologic production of IFNγ following ip. administration of IL-12 demonstrating a successful BM engraftment. By contrast, IL-12Rβ2^−/−^ recipients that were transplanted with BM from syngeneic IL-12Rβ2^−/−^ did not respond to IL-12 administration. Statistical analysis (seven mice/group) was carried out by (ANOVA) Wilcoxon rank test. (*) indicates statistical difference *p* < 0.05.

**Figure 5:**
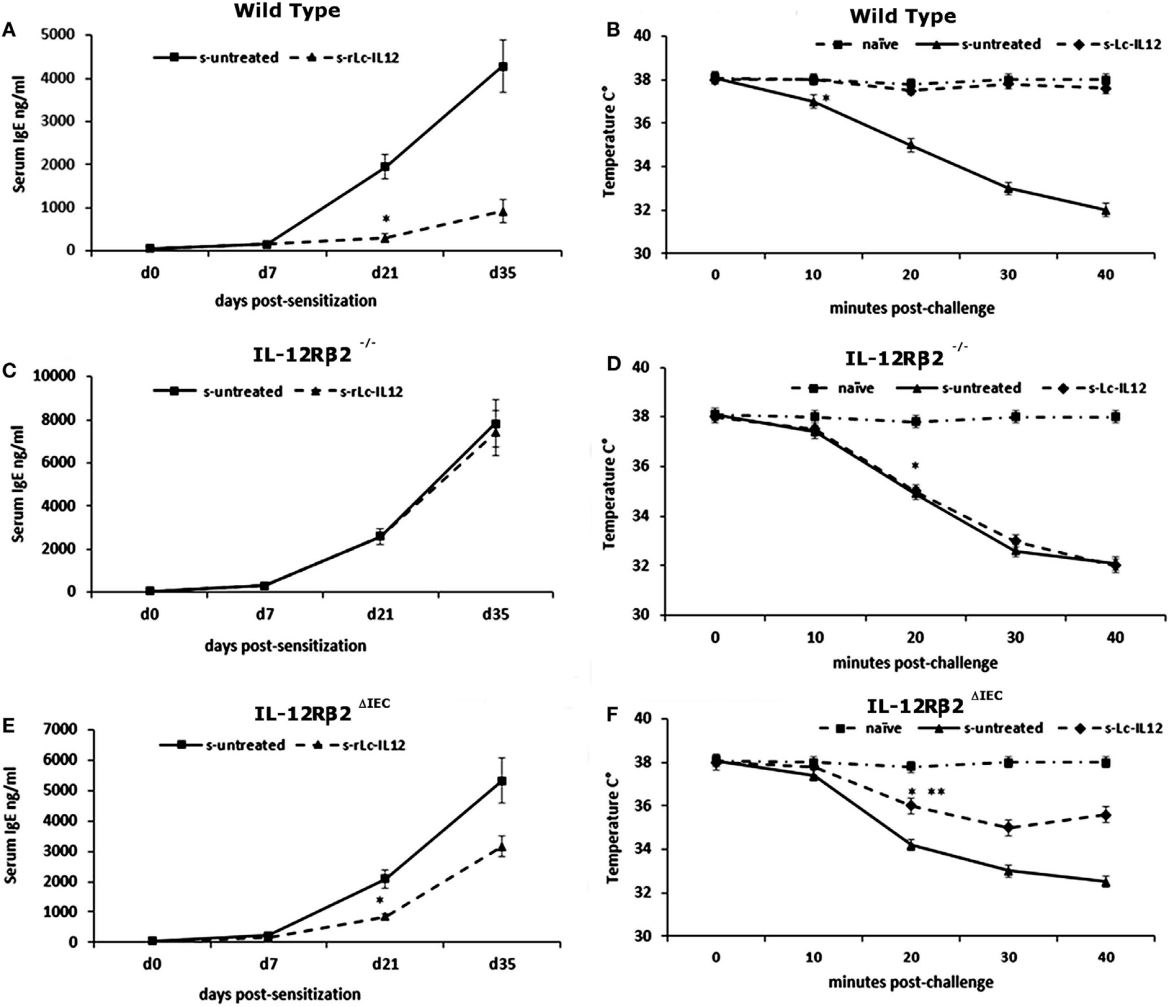
Effects of oral delivery of rLc-IL12 on IgE levels and body temperature in sensitized wild-type (wt) and chimeric mice. Administration of rLc-IL12 by oral gavage in a single dose to wt mice 24 and 2 h before administration of the sensitizing mixture significantly reduced the levels of allergen (peanut)-specific IgE antibody **(A)** and prevented the drop of the body temperature as assessed by rectal probe **(B)** showing that oral delivery of IL-12 *via* a genetically engineered strain of *Lactococcu lactis* could suppress food allergic reaction by restoring appropriate levels of IL-12. By contrast, administration of IL-12 in chimeric IL12Rβ2^−/−^ mice (BM transplant from IL-12Rβ2^−/−^→IL-12Rβ2^−/−^) had no effect. Indeed, neither plasma levels of IgE nor body temperature were affected by treatment with rLc-IL12 **(C,D)**. The pattern was different in chimeric IL-12Rβ2^∆IEC^ mice (BM transplant from WT→IL-12Rβ2^−/−^) that lacked the IL-12Rβ2 exclusively on intestinal epithelial cells (IECs) **(E,F)**. In these mice, oral delivery of rLc-IL12 afforded a reduced protection against food allergy. Administration of rLc-IL-12 significantly reduced plasma levels of IgE compared to rLc-IL12 untreated sensitized mice; however, the extent of the reduction was much less pronounced compared to what was observed in fully competent IL-12Rβ2 mice. Similarly, oral administration of rLc-IL12 did not completely abolished the allergy-associated drop in body temperature although the drop was significantly (**) less pronounced compared to sensitized rLc-IL12 untreated mice. Statistical analysis (8–10 mice group) was carried out by (ANOVA) Wilcoxon rank test. (*, **) indicates statistical differences. *p* < 0.05.

**Figure 6:**
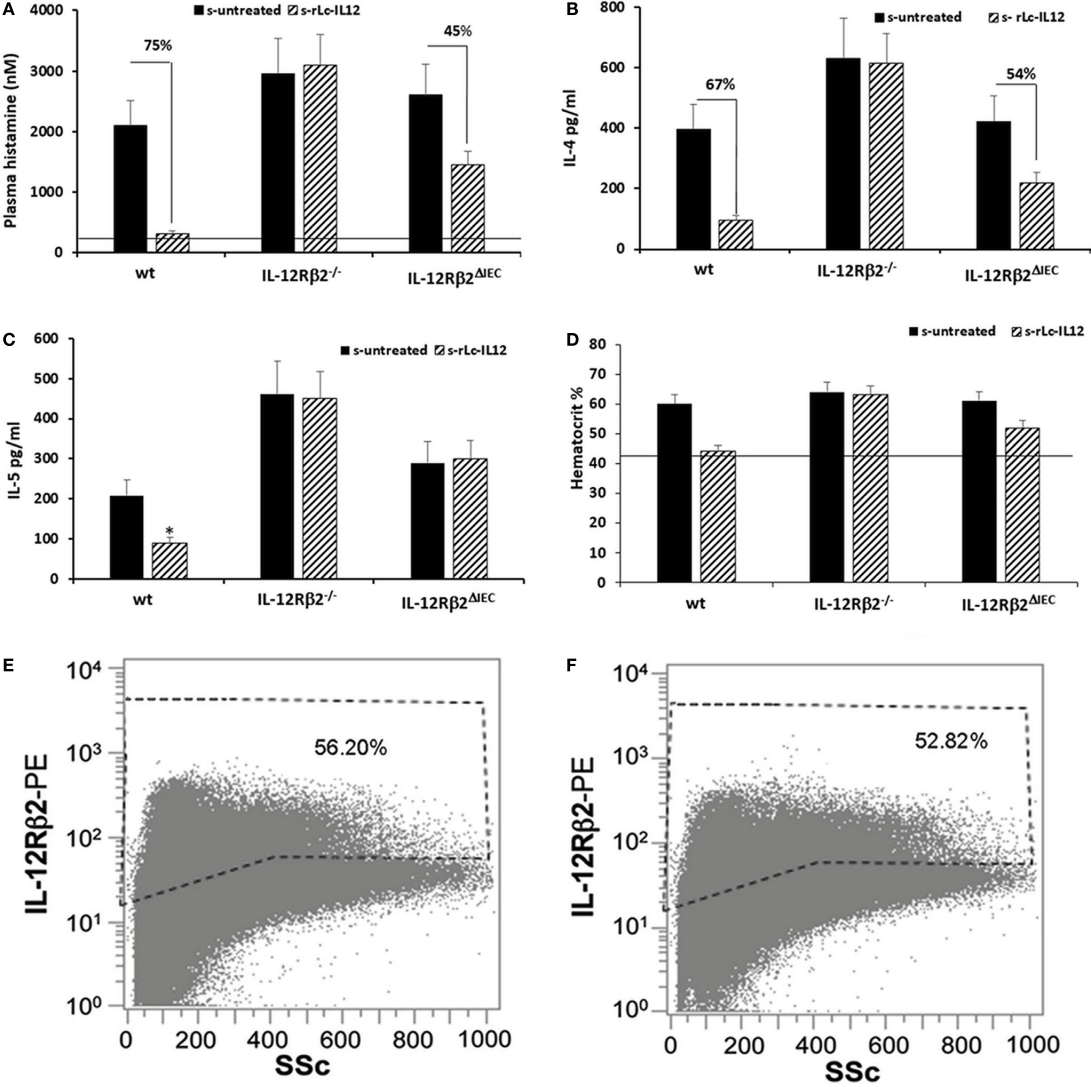
Efficacy of rLc-IL12 on additional parameters of food allergy in sensitized wild-type (wt) and chimeric mice. Oral administration of IL-12 reduced levels of plasma histamine by 75% in sensitized wt mice compared to IL-12 untreated syngeneic mice **(A)**. By contrast, no effects were seen in chimeric IL-12Rβ2^−/−^ mice; instead the IL-12 mediated reduction of plasma histamine in chimeric IL-12Rβ2^∆IEC^ totaled only to 45%. Also, levels of IL-4 produced by splenocytes following *in vitro* recall allergen challenge showed a similar pattern **(B)**. Production of IL-4 in wt mice treated with rLc-IL12 dropped 67% compared to sensitized untreated mice; the same treatment was not effective in chimeric IL-12Rβ2^−/−^ mice and only partially effective in IL-12Rβ2^∆IEC^ mice (54%). By contrast, levels of IL-5 following *in vitro* recall allergen challenge were reduced by rLc-IL12 only in wt but not in either chimeric mouse variants **(C)**. Hematocrit was evaluated as a measure of vascular leakage **(D)**. Administration of rLc-IL12 restored near physiological levels of hematocrit in wt mice; no effects were seen in chimeric IL-12Rβ2^−/−^ mice and only partial restoration of the hematocrit was seen in chimeric IL-12Rβ2^∆IEC^. Finally, representative histograms showed that levels of IL-12Rβ2 on intestinal epithelial cells (IECs) did not vary between sensitized **(F)** and naïve mice **(E)**. Statistical analysis (8–10 mice group) was carried out by (ANOVA) Wilcoxon rank test. *p < 0.05.

To test the biological role of IEC-associated IL-12Rβ2 chain, we analyzed the ability of a recombinant bacterial vector secreting bioactive IL-12p70 (rLc-IL12) (12) to suppressing food allergic response in wt and chimeric IL-12Rβ2^−/−^ and IL-12Rβ2^∆IEC^ mice that lack IL-12Rβ2 on IECs. First, we assessed the production of IFNγ in response to systemic administration of IL-12 in IL-12Rβ2^−/−^ mice following BM transplant from wt mice (WT→IL-12Rβ2^−/−^) at week 8 posttransplant. (Figure 4) In agreement with a previous report (15), the presence of an intact IL-12R enabled wt mice to produce *in vivo* significant amount of IFNγ following i.p. administration of IL-12. By contrast, IL-12Rβ2^−/−^ and chimeric IL-12Rβ2^−/−^ (IL-12Rβ2^−/−^→IL-12Rβ2^−/−^) mice did not produce detectable levels of IFNγ. However, IFNγ production was completely restored in IL-12Rβ2^−/−^ mice by BM transplant from wt mice (WT→IL-12Rβ2^−/−^). The result of the functional assay was paralleled by the observation that post-BM transplant mice displayed similar levels of MHCII^+^ cells in the lp compared to wt mice (Figure S2 in Supplementary Material). Similar results were obtained at week 28 posttransplant (not shown). Taken together these data show a successful BM engraftment. Subsequently we investigated the role of IEC-associated IL-12Rβ2 in the regulation of immune response using a well-established mouse model of food allergy. The IL-12 secreting bacterial vector rLc-IL12 was delivered prior to each administration of the sensitizing mixture and its effects on food allergic reaction were monitored upon final allergen challenge by monitoring a panel of allergic symptoms. Oral delivery of rLc-IL12 significantly reduced plasma levels of IgE in sensitized wt mice compared to sensitized untreated mice (Figure 5A). Also, in these mice we observed that rLc-IL12 prevented the drop in temperature associated with allergic reaction following the final challenge with the causal allergen (Figure 5B). By contrast, IgE response was not affected by the administration of rLc-IL12 in chimeric IL-12Rβ2^−/−^ mice (Figure 5C); also, these mice showed a markedly higher production of IgE compared to wt throughout the allergen sensitization period (summary in Table 1). In this group of mice, rLc-IL12 also failed to prevent the allergy-associated drop in body temperature (Figure 5D). The pattern in IL-12Rβ2^∆IEC^ appeared to be different. In this group, the administration of rLc-IL12 significantly reduced plasma levels of IgE but the magnitude of the reduction was smaller compared to wt mice (41 and 93%, respectively) (Figure 5E). A similar profile was observed in regard to the effects of rLc-IL12 on body temperature (Figure 5F). Here too the treatment with rLc-IL12 significantly prevented the drop of body temperature observed in wt mice but not as much as observed in the wt cohort. A similar trend was observed for all the other parameters of Type I allergic reactions monitored (Figure 6). Plasma levels of histamine in sensitized wt mice reached 2,100 ± 185 ng/ml and were reduced to 320 ± 21 ng/ml (*p* < 0.01) in rLc-IL12-treated mice leading to a 75% reduction (Figure 6A). Furthermore, while no effects were seen on histamine levels in IL-12Rβ2^−/−^ mice, the administration of rLc-IL12 led to a reduction of plasma levels in IL-12Rβ2^∆IEC^ from 2,600 ± 212 to 1,450 ± 104 ng ml totaling to a 45% reduction. Treatment with rLc-IL12 also affected IL-4 production following *in vitro* recall antigen challenge of splenocytes in both wt and chimeric IL-12Rβ2^∆IEC^ but not in chimeric IL-12Rβ2^−/−^ mice. Levels of IL-4 in culture supernatants following splenocytes challenge with the causal allergen were significantly reduced (*p* < 0.01) by treatment with rLc-IL12 (Figure 6B). By contrast, production of IL-5 was downregulated by rLc-IL12 only in wt mice but not in the two chimeric groups (Figure 6C). Hematocrit was utilized to monitor vascular permeability leakage, an additional feature of type I hypersensitivity reaction (Figure 6D). Hematocrit levels were increased in sensitized untreated wt mice (60.5%) compared to baseline levels (41.5%) and, in these mice was significantly reduced by rLc-IL12 (43.5%). Administration of rLc-IL12 did not suppress vascular leakage in chimeric IL-12Rβ2^−/−^ mice, however, rLc-IL12 treatment reduced hematocrit levels in chimeric IL-12Rβ2^∆IEC^ mice compared to untreated syngeneic mice (42.2% and 61.2%, respectively). Finally, we observed that the expression of the IL-12Rβ2 on IECs did not differ between sensitized and control naïve wt mice (Figures 6E,F) suggesting that allergy-associated skewed T_H_1/T_H_2 ratio did not affect the expression of the IL-12Rβ2 chain on IECs.

**Table 1:** Effects of rLC-IL12 on IgE production (ng/ml): day 35.

Group (*n* = 8/10) reduction	Control	s-Untreated	s-rLC-IL12	*p*	%
Group I (wt)	<10	4,280 ± 950	275 ± 72	<0.001	93.6
Group II (IL-12Rβ2^−/−^)	<10	7,820 ± 1,050	7,250 ± 1,220	>1	8
Group III (IL-12Rβ2^βIEC^)	<10	5,320 ± 980	3,150 ± 650	0.05	41

*Levels of plasma IgE were evaluated in wt (Group I) and chimeric mice deficient for IL-12Rβ2 chain (Group II, IL-12Rβ2^−/−^) or deficient for IL-12Rβ2 chain in intestinal epithelial cells (Group III, IL-12Rβ2^∆IEC^)*.

*Values refer to levels of IgE prior to the beginning of the sensitization period (control) and in mice sensitized and left untreated (s-untreated) or treated with rLC-IL12 (s-rLC-IL12) on day 35 following the final antigenic challenge*.

### Downregulation of IEC-Derived TSLP Following Oral Delivery of rLc-IL12 Did Not Occur Directly *via* IEC-Associated IL-12Rβ2

**Figure 7:**
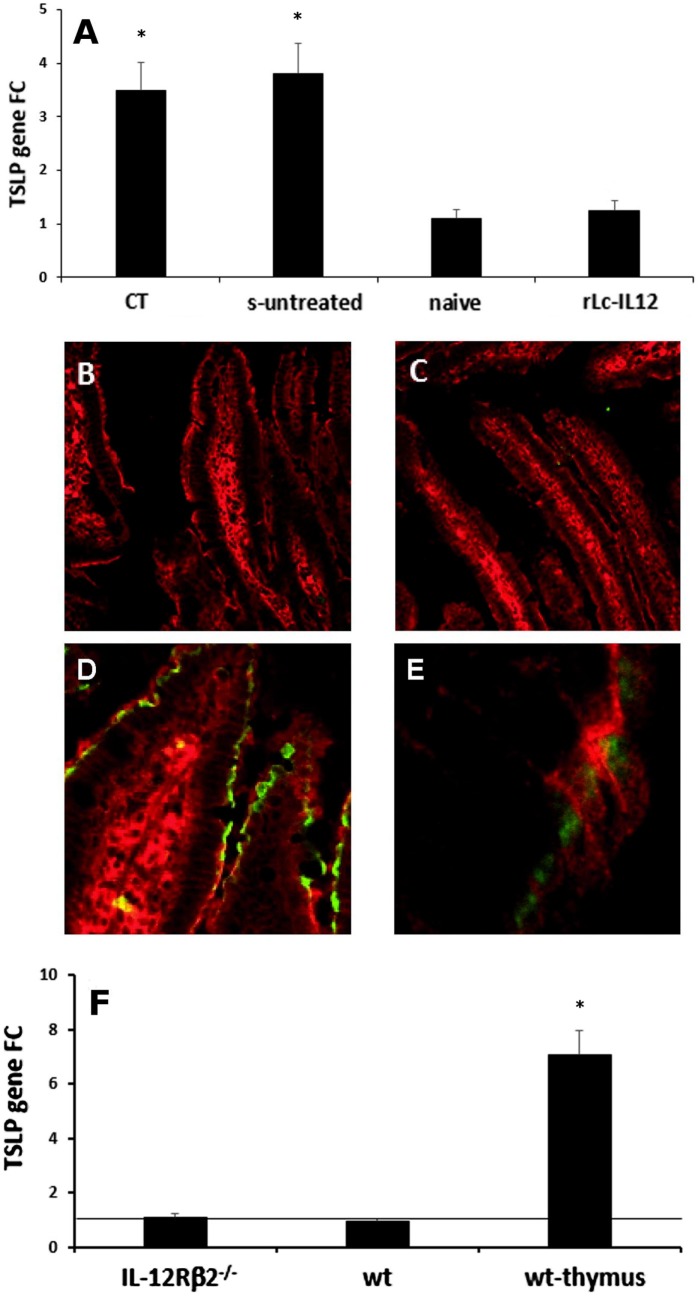
IL-12-mediated downregulation of IEC-derived TSLP does not occur directly *via* IEC-associated IL-12Rβ2. RT-PCR analysis of the gut epithelium **(A)** showed that oral delivery of the adjuvant cholera toxin (CT) induced a significant increase of TSLP in the gut epithelium similar to that observed following delivery of the complete sensitizing mixture (s-untreated) compared to control mice (naive). Allergy-associated levels of TSLP were abrogated by treatment with rLc-IL12. This notion was further confirmed by immunohistochemistry. Immunofluorescence analysis with anti-TSLP antibody did not detect levels of TSLP in the IECs of both control **(B)** and rLc-IL12-treated mice **(C)**. In contrast, specific anti-TSLP staining (green) was observed in the epithelium of sensitized rLc-IL-untreated mice **(D)**, detail in **(E)**. Sections were counter-stained with antiactin antibody (red). Finally, expression of TSLP gene in the intestinal epithelium of naïve IL-12Rβ2^−/−^ mice **(F)** showed only a minor increase that did not reach statistical significance compared to wt syngeneic mice. In this case, the thymus from wt mice was used as positive control. Transcript analysis was carried out in replicate mice (four to six mice/group) in two independent assays. Data were analyzed using a Student’s *t*-test followed by application of the Bonferroni correction the level of which was set at *p* ≤ 0.0125. (*) indicate statistical difference. *p* < 0.05.

The overexpression of the IEC-derived cytokines TSLP and IL-33 is critical in food allergy (23–25). We then tested the hypothesis that the engagement of IL-12 with the functional IEC-associated IL12Rβ2 chain could play a role in the regulation of these proallergic cytokines. First, quantitative RT-PCR analysis (Figure 7A) showed that TSLP gene expression was significantly upregulated in the gut epithelium of sensitized mice as well as in mice treated with CT alone, compared to control non-sensitized (PBS-treated) mice. Furthermore, administration of rLc-IL12 led to a significant downregulation of TSLP gene expression in the gut epithelium of sensitized mice. The regulatory effect of rLc-IL12 on IEC-derived TSLP was further confirmed by immunohistochemistry. The latter approach did not detect TSLP neither in PBS- nor in rLc-IL12-treated mice (Figures 7B,C) but only in sensitized mice (Figures 7D,E). Taken together, these results concurred with reports that suggested a critical role of the epithelium-derived TSLP in allergic reactions (23, 24) and pointed to an important role of IL-12 in the regulation of the epithelium-derived TSLP. Furthermore, immunofluorescence staining was carried out in both IL-12Rβ2^−/−^ and IL-12Rβ2^∆IEC^ chimeric mice undergoing allergic sensitization and treated with rLc-IL12. Both sensitized chimeric mice retained the ability to produce TSLP by IECs (Figure S3 in Supplementary material). The latter observation further suggested that IEC-associated IL-12Rβ2 plays a role in the control of TSLP production by IECs. However, when the expression of TSLP was assessed in the epithelium of naïve IL-12Rβ2^−/−^ mice we did not observe an increase in the level of TSLP compared to naïve wt counterparts (Figure 7F). Taken together these data point to a scenario suggesting that IEC-associated IL-12Rβ2 is an important, but not the only factor controlling TSLP production by IECs. Overall, taken together these data demonstrated that the downregulation of IEC-derived TSLP following oral administration of IL-12 is not directly linked to IL-12 engagement with the IL-12Rβ2 chain on IECs, thus suggesting that IL-12 might trigger the production of additional factor(s) that in turn controlled the production of TSLP by the intestinal epithelium. Finally, as a corollary parallel analysis showed that administration of rLc-IL12 suppressed allergic reaction without downregulating levels of IEC-derived IL-33 while at the same time partially suppressing allergy-associated expression of OX40L (Figure S4 in Supplementary Material). The latter observation pointed to a synergistic action of TSLP and IL-33 on the expression of the allergy-critical OX40L rather than an exclusive role of IL-33 as previously suggested (25).

## Discussion

Here, we report that mouse IECs expressed the IL-12Rβ2 but not the complementary IL-12Rβ1 chain that functions as an incomplete, yet functional IL-12R that plays a role in lympho-epithelial cross talk. In the mouse system, the two β1 and β2 components of the IL-12R have specific role in IL-12-mediated binding and signaling. IL-12Rβ1 chain is the primary binding site conferring both high and low affinity, instead the IL-12Rβ2 only binds IL-12 with low affinity (26). However, these studies carried out in IL-12Rβ1^−/−^ mice initially suggested that the β1 component was required for both high affinity binding and for immune cells response to IL-12 it was later showed that only the IL-12Rβ2 chain was strictly required for IL-12 signaling (15). Indeed, no IL-12 mediated biological functions could be detected in IL-12Rβ1^+^/β2^−^ mice. Combined, these studies suggested that in the mouse system the IL-12Rβ1 is primarily responsible for binding and IL-12Rβ2 is further required for IL-12 signaling. Our study extends this concept showing that while this is the case for immune cells the expression of the sole β2 chain of the IL-12R on IECs might be an important component of the lymphoepithelial cross talk.

The expression of IL-12Rβ2 on IECs depended on immune–derived stimuli. This concept stemmed from several observations. A mouse small-intestine derived epithelial cell line and primary intestinal organoids cultured in the absence of gut-derived immune cells did not express the IL-12Rβ2 chain. By contrast, the presence of gut-derived immune cells triggered the expression of the IL-12Rβ2 subunit by both an IEC line and primary intestinal organoids. This is in line with previous reports showing that the expression of the IL-12Rβ2 but not of the IL-12Rβ1 is regulated by signals (i.e., cytokines) originating from the immune system (22). Although the nature of the signals remains to be determined it appeared that both soluble factor(s) and cell-to-cell interaction are important to trigger the expression of IL-12Rβ2. This was also suggested by the observation that *in vivo*, the expression of the IL-12Rβ2 chain was located at the basolateral domain of IECs at the interface with local lp-immune cells.

IL-12 ligation with the conventional IL-12Rβ1/β2 complex signaled through the Janus kinase (JAK)-signal transduce pathway in both human and mouse (27, 28); in particular, the critical role of Stat-4 in IL-12R signaling has been demonstrated *in vivo via* the use of Stat 4-deficient mice (29). On the other hand, although in IL-12Rβ1-deficient humans the presence of the sole IL-12Rβ2 chain was not sufficient to confer full IL-12 responsiveness *via* JAK-mediated pathway, T cells from these individuals could still develop into T_H_1 cells and respond to IL-12 (22). In this case, IL-12 signaling occurred *via* an IL-12Rβ1-independent pathway that involved MAPK pathways. In agreement with these observations, we reported that IL-12/IL-12Rβ2 ligation on freshly isolated IL-12Rβ1^−^/β2^+^ IECs induced activation of phopho-MAPKp38 in a manner similar to that obtained by a non–specific MAPK activator. At present the possibility that other surface molecules might be associated with IL-12Rβ2 on IECs to form an additional variant of the IL-12R that operates *via* MAPK cannot be ruled out; however, our results, strongly suggested that the presence of IL-12Rβ2 alone on IECs is sufficient to confer responsiveness to IL-12.

The presence of a functional, albeit incomplete IL-12R on IECs suggested that the IL-12/IL-12Rβ2 ligation could be important in controlling the production by IEC-derived cytokines. We tested this hypothesis in a mouse model of food allergy. Its utilization was prompted by the notions that in this model the allergic sensitization to food components (peanuts) was characterized by the lack of IL-12 in the gut but not in the systemic immune system (10); in addition, using the same model others have previously shown that oral delivery of liposome encapsulated recombinant IL-12 suppressed allergic reaction (11). We report here that in chimeric mice the lack of IEC-associated IL-12Rβ2 significantly reduced but not completely abolished the antiallergic capability of a *L. lactis* strain genetically engineered to secrete bioactive IL-12. This result suggested that IL-12 delivered orally *via* the recombinant bacterial vector produced the antiallergic activity by engaging a variety of IL-12 responding cells either locally or systemically that included IECs. This result could not be attributed to an unsuccessful BM engraftment in IL-12Rβ2^−/−^ recipients; in these mice the production of IFNγ following systemic administration of IL-12 was completely restored at 8 weeks after transplant of BM from wt mice.

Oral delivery of Lc-IL12 had a significant impact on the levels of TSLP produced by IECs in sensitized mice. The production of TSLP by IECs is an important example of the importance of the tight regulation of the finely tuned cytokine network in the gut. On one hand, TSLP plays a critical role in maintaining the intestinal immune homeostasis by shaping the anti-inflammatory features of dendritic cells (2) and Treg (30); on the other, strong evidence suggested that its over-expression is instrumental for the development of allergic reactions (23, 24). We found, in agreement with another report (25) that mice undergoing the sensitization protocol employed in our study displayed a significant increase of TSLP production by IECs during and following the sensitizing procedure. However, oral administration of rLc-IL12 significantly downregulated TSLP production by IECs while at the same time supressing allergic symptoms. The action of IL-12 on TSLP production however did not appear to be directly linked to the IL-12/IL-12R ligation on IECs. Naive IL-12Rβ2^−/−^ mice showed only a slight increase of IEC-derived TSLP, thus suggesting that the production of TSLP by IECs is under the control of multiple factors. It is known that the production of certain cytokines, such as IL-13 required the simultaneous presence of two cytokines (i.e., IL-33 and IL-7) (31) and this notion lends support to the hypothesis that a dual signal might be required also to control the production of TSLP by IECs. This would suggest that IL-12 mediated regulation of TSLP production required the synergy with factor(s) that are suppressed/downregulated in sensitized mice but that are still retained in non-sensitized IL-12Rβ2^−/−^ mice. Overall, our study suggested that IL-12 and TSLP are part of an important regulatory cytokine loop operating at the interface between the intestinal epithelium and the underlying immune system that ultimately plays a role on the host’s ability to mount appropriate immune responses. Indeed, it has been shown previously that TSLP directly suppressed the production of IL-12 in DCs (32); here, we report that in turn IL-12, in combination with other factors plays a role in controlling the production of TSLP by IECs.

The utilization of a mouse model of food allergy to understand the biological role of IL-12Rβ2 in controlling the production of IEC-derived cytokines led to the parallel finding that the suppression of food allergy by IL-12 was indirectly linked to the downregulation of TSLP but did not affect levels of another allergy-critical epithelium-derived cytokine, IL-33. Instead, at the same time oral delivery of IL-12 partially suppressed allergy-associated expression of OX40L. This was rather surprising considering that IL-33 but not TSLP was previously indicated as having a critical, non-redundant role in allergy (25). Thus, our work in agreement with a recent report (33) argued against the concept that the alteration of a single immunoregulatory pathway (i.e., IL-33) is at the core of allergic reactions. Our data point to a synergistic action of TSLP and IL-33 on OX40L expression, as also more recently shown in a rhinovirus-mediated allergic reaction to aeroallergen (34), rather than an exclusive IL-33-mediated effect as previously suggested (25). In summary, taken together these data show that IL-12Rβ2 chain functions as an incomplete yet functional IL-12R on IECs and overall further highlight the importance of a finely tuned lymphoepithelial cross talk in the intestine in maintaining health.

## Ethics Statement

Experiments were approved by the University of East Anglia Animal Welfare and Ethical Review Body (AWERB; ref: 70/7583) and conducted under the guidelines of the Scientific Procedure Animal Act (1986) of the United Kingdom. Also, experimental protocols were approved by the Animal Care and Use Committee of the University of Siena under the “Guiding Principles for Research Involving Animals and Human Beings.”

## Author Contributions

CN, AN, MG, EB and MR designed the study and wrote the manuscript; MR, AM, NG, AD, KI, AP, ML, JJVB, GM and US performed the experiments, provided technical support and carried out data analysis; CN, AN and EB supervised research.

## Conflict of Interest Statement

The authors declare that the research was conducted in the absence of any commercial or financial relationships that could be construed as a potential conflict of interest.
