# Supplementary material for: Morphological and Functional Characterization of IL-12Rβ2 Chain on Intestinal Epithelial Cells: Implications for Local and Systemic Immunoregulation
Source: Front Immunol. 2018 May 29;9:1177. doi: 10.3389/fimmu.2018.01177 (PMC5986899; doi:10.3389/fimmu.2018.01177)
Supplement: Supplementary file 1 [file image_1.PDF]

Supplementary Material

Morphological and functional characterization of IL-12Rβ2 chain on intestinal epithelial cells: implications for local and systemic immunoregulation

Mari Regoli, Angela Man, Nadhezda Gicheva, Antonio Dumont, Kamal Ivory, Alessandra Pacini, Gabriele Morucci, Jacopo JV Branca, Monica Lucattelli, Ugo Santosuosso, Arjan Narbad, Massimo Gulisano, Eugenio Bertelli, Claudio Nicoletti*

*** Correspondence:** claudio.nicoletti@unifi.it

1 Supplementary Figures and Tables

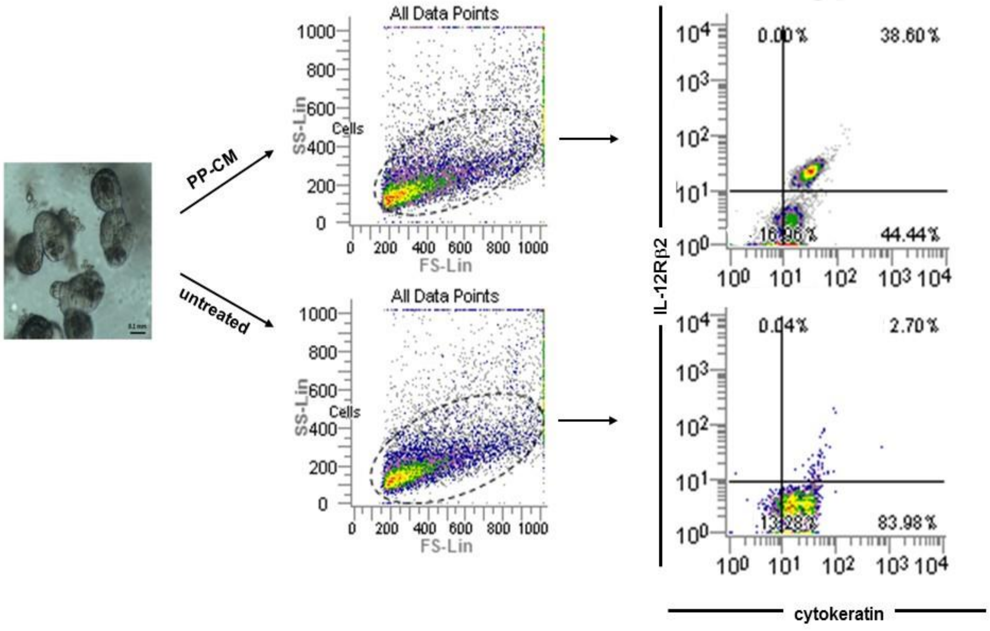

Supplementary Figure 1. **Expression of IEC-associated IL-12Rβ2 chain is regulated by signals derived from immune cells.** Primary intestinal organoids were developed *in vitro*. In order to evaluate the effects of immune system-derived signals on the expression of the IL-12Rβ2 chain on IECs culture

medium was supplemented with conditioned medium collected from 3-day culture of Peyer's patch-derived immune cells (PP-CM) or left not supplemented (untreated). Following the preparation of cells suspension the cells were analyzed by flow cytometry. Organoid intestinal epithelial cells grown in the presence of PP-CM showed a significantly higher proportion of organoid cells expressing the IL-12Rβ2 chain compared to cell grown in normal not supplemented culture medium. Furthermore, the effect of the PP-CM is twofold. Indeed, in addition to the increase of the percentage of cells expressing the IL-12Rβ2 chain, these cells also showed an increased expression of the epithelial marker cytokeratin. Two batches of intestinal organoids/treatment were prepared and samples were run in triplicate.

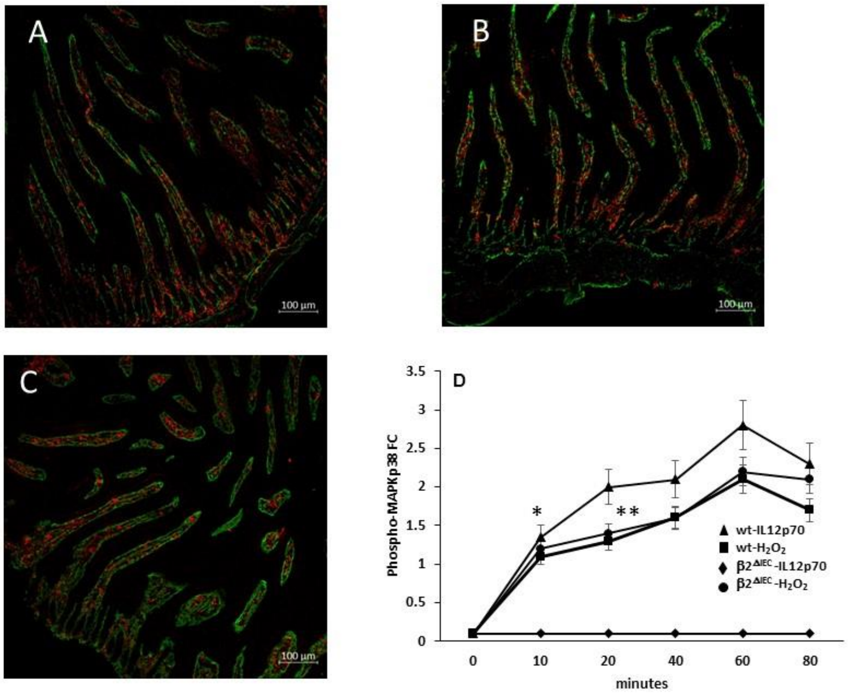

Supplementary Figure 2. **Post BM-transplant levels of MHC II$^+$ cells in the intestinal tissue**. Wild type (A) and chimeric IL-12Rβ2$^{-/-}$ (BM from IL-12Rβ2$^{-/-}$ → IL-12Rβ2$^{-/-}$) recipient mice) (B) and IL-12Rβ2$^{\Delta IEC}$ (BM from wt → IL-12Rβ2$^{-/-}$ recipient mice) (C) were monitored for the levels of MHCII$^+$ cells (red) using rat anti-I-A/I-E(clone M5/114.15.2, Biolegend) within the lamina propria (lp) on week 8 post BM-transplant. Sections were counterstained (green) with rabbit anti-entactin antibody (Abcam). Immunofluorescence analysis revealed similar levels of expression of MHCII$^+$cells in the lp thus showing a successful engraftment. These analysis complemented the functional experiment carried out as described in Figure 4 of the main text. Furthermore, IECs from both wild type and IL-12Rβ2$^{\Delta IEC}$ mice were isolated and challenged *in vitro* with bioactive IL-12p70; levels of phospho-MAPKp38 were then evaluated by flow cytometry. In agreement with what observed in Figure 1F for wt and IL-12Rβ2$^{-/-}$ mice

we observed that also IECs from chimeric IL-12R$\beta 2^{\Delta IEC}$ ($\beta 2^{\Delta IEC}$-IL12p70) did not respond to IL-12. By contrast, these cells responded when exposed to a non-specific activation stimulus (H_2O_2). (*) indicates statistical difference between wt-derived IEC challenged with both IL-12p70 or H_2O_2 and $\beta 2^{\Delta IEC}$-derived IEC challenged with H_2O_2 compared to $\beta 2^{\Delta IEC}$-derived IECs challenged with IL-12p70. (**) indicates statistical difference between wt-derived IECs challenged with IL-12p70 and both wt- and $\beta 2^{\Delta IEC}$-IECs challenged with H_2O_2. Experiments were performed in triplicate using freshly isolated IECs from 3 mice/group.

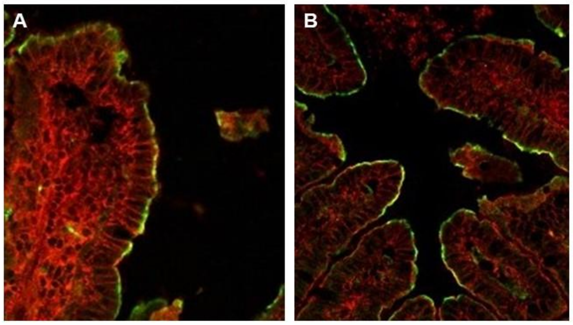

Supplementary Figure 3. Immunofluorescence staining with anti-TSLP antibody in the gut of chimeric mice. Immunofluorescence staining carried out in both IL-12R$\beta 2^{-/-}$ (A) and IL-12R$\beta 2^{\Delta IEC}$ (B) chimeric mice undergoing allergic sensitization showed detectable levels of TSLP (green) in the epithelium following oral delivery of recombinant bacterial vector producing IL-12p70 (rLc-IL12). Sections were counterstained (red) with mAb anti-β-actin antibody (Clone AC15) (Sigma-Aldrich,

Milan, Italy). These data complemented data in Figure 7B-E of the main text and confirm that the presence of IL-12Rβ2 plays a role, among other factors in the control of TSLP production by IECs**.**

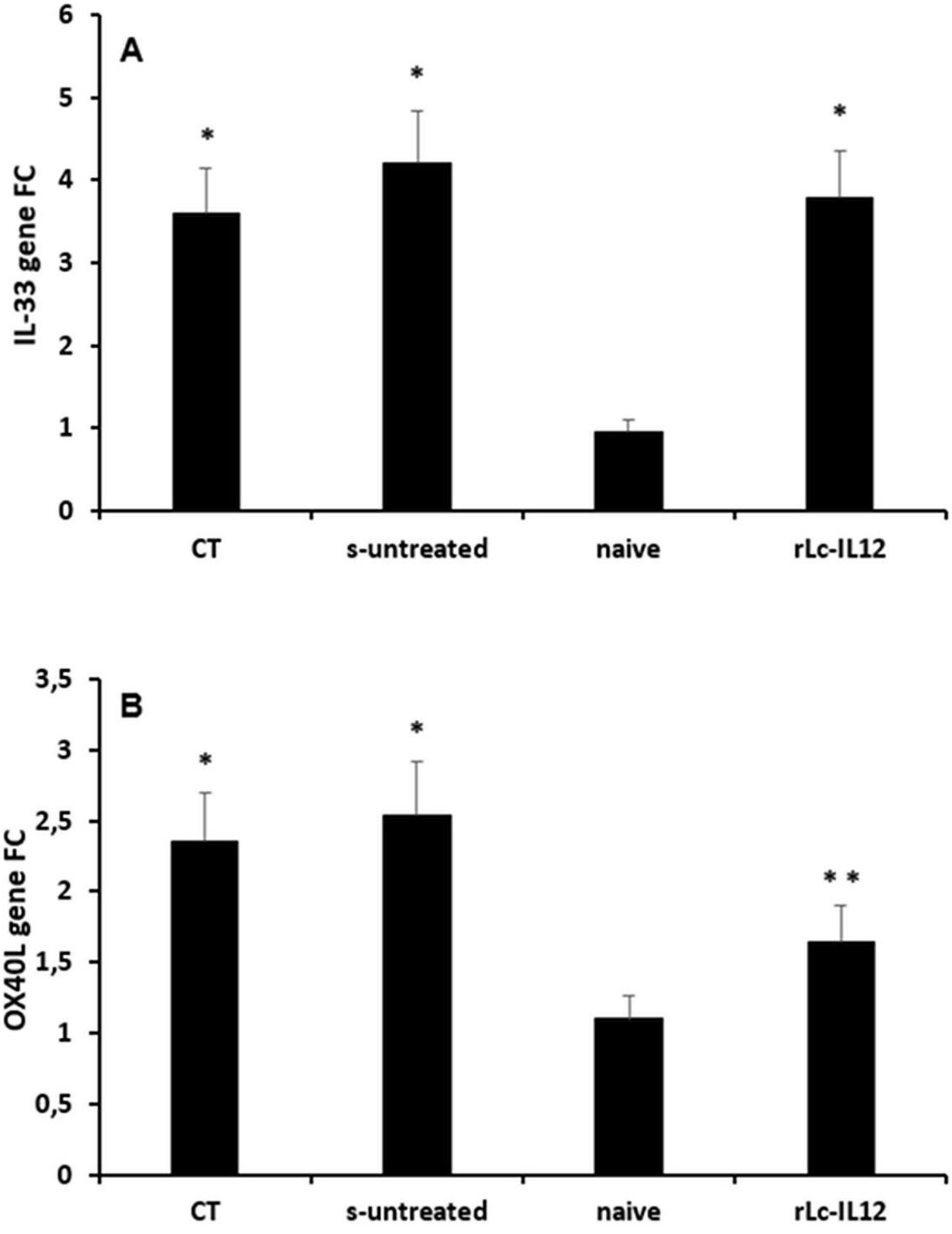

Supplementary Figure 4. Effects of rLc-IL12 on the intestinal expression of IL-33 and OX-40L. Mice treated with cholera toxin (CT) and in those sensitized by administration of CT in combination with allergen (s-untreated) and not treated with rLc-IL12 induced a significant increase of the expression of IL-33 in the intestinal epithelium compared to control naive mice; further, levels of IL-33 were not

affected by treatment with rLc-IL12 (A). Furthermore (B), also intestinal levels of OX40L were significantly up-regulated in CT and sensitized (s-untreated) mice compared to control naïve (*); however, in this case oral administration of rLc-IL12 significantly down-regulated the expression of OX40L but not completely abolished it and it remained significantly higher than in naïve mice (**) . Transcript analysis was carried out in replicate mice (4-6 mice/group) in two independent assays. Data was analysed using a Student's t-test followed by application of the Bonferroni correction the level of which was set at $p \leq 0.0125$. $P<0.05$.

frontiers
